# Evaluation of Oral Ginger Efficacy against Postoperative Nausea and Vomiting: A Randomized, Double - Blinded Clinical Trial

**DOI:** 10.5812/ircmj.12268

**Published:** 2013-12-05

**Authors:** Akram Sadat Montazeri, Azam Hamidzadeh, Mehdi Raei, Malihe Mohammadiun, Azam Sadat Montazeri, Reza Mirshahi, Hosein Rohani

**Affiliations:** 1 Center for Health-Related Social and Behavioral Sciences Research, Shahroud University of Medical Sciences, Shahroud, IR Iran; 2 School of Nursing & Midwifery, Shahroud University of Medical Sciences, Shahroud, IR Iran; 3 Clinical Research Development Center, Qom University of Medical Sciences, Qom, IR Iran; 4 School of Civil and Architectural Engineering, Shahrood University of Technology, Shahrood, IR Iran; 5 School of Pharmacy, Tehran University of Medical Sciences, Tehran, IR Iran; 6 School of Medicine, Tehran University of Medical Sciences, Tehran, IR Iran; 7 School of Health, Isfahan University of Medical Sciences, Isfahan, IR Iran

**Keywords:** Ginger, Nausea, Vomiting

## Abstract

**Background:**

Postoperative nausea and vomiting is one of the most common side effects associated with surgical procedures.

**Objectives:**

The aim of this study was to determine the effect of ginger on intensity of nausea and vomiting after surgical procedures.

**Patients and Methods:**

This study was a randomized, double blinded, clinical trial. 160 eligible patients were randomly assigned into experimental or placebo groups. The experimental group received 4 capsules containing 250 mg ginger and placebo group received 4 placebo capsules 1 hour before surgery. The severity of nausea and vomiting was measured at 2, 4, 6 hours post operation using visual analogue scale and a structured questionnaire. The data were analyzed by independent t - test, Mann-Whitney U test, chi –square and GEE using SPSS 16 and STATA version 11.

**Results:**

Mean nausea score at 2 hours post operation was significantly lower in the experimental group (P= 0.04). Mean nausea score at 4 and 6 hours post operation was lower in the experimental group; however, there was no significant difference between the groups at any time post operation. The frequencies of nausea in the experimental group at 2 and 6 hours post operation were lower than that in the placebo group, however, at 2 hours post operation, it was borderline significant (P = 0.05) There was no significant differences between two group in the intensity of vomiting at any time.

**Conclusions:**

Use of ginger was effective at decreasing postoperative nausea. Ginger could be used as a safe antiemetic drug at post operation.

## 1. Background

Surgery and anesthesia, associated with innovation of recent drugs and methods, resulted in advancement in patient care. However, these methods have some side effects including Intestinal paralysis, ileus, nausea, and vomiting (1). In addition the anesthesia type, type of the surgery, narcotic drugs use, sex, hypotension and physical condition of the patient, have great effects on the development of nausea and vomiting (1).

Postoperative Nausea and vomiting is one of the most common complications of surgery, and usually occurs after any type of anesthesia (2). This complication may occur up to 24 hours after the surgery and in 20-30% of patients. 70-80% of these patients experience severe nausea and vomiting. Patients, who have a history of nausea and vomiting following surgery, believe that this side effect is the most stressful complication after the surgery. Most of them prefer to suffer severe pain after the surgery instead of nausea and vomiting (1, 3). Uncontrolled nausea and vomiting, leads to delay in patient’s discharge, increased treatment costs, and decreased patient satisfaction (2).

Nausea and vomiting after a surgery leads to dehydration, electrolyte disorders, hypertension, traction sutures, increased bleeding from skin flops, and finally delay in patient’s discharge. This complication can increase the risk of pulmonary aspiration if the airway reflexes have been decreased due to the residual effects of anesthetic drugs (1, 3-5).

Since about 50 years ago, numerous drugs have been known for prevention and treatment of nausea and vomiting. The most commonly used drugs to relieve nausea and vomiting are Metoclopramid and Droperidol , which have limited administration because of their side effects including unawareness of time and location, extrapyramidal symptoms, cardiovascular complications, postural hypotension, drowsiness, akathisia, elevated liver enzymes, and agranulocytosis (6). Experience has shown that synthetic drugs have too many inappropriate site effects despite of their effectiveness (7). Today several methods are applied to control the nausea and vomiting, medications and complementary therapies are some of them .Selection and prescription of appropriate pharmaceutical or non-pharmacologic therapy leads to improvements in life style, performance of patients and desired effects (8). Thus complementary and alternative therapies may be applied to control complications, separately or in association with standard methods (9, 10).

Traditional and complementary medicine has lots of positive points including variety, flexibility, easy access, availability in many parts of the world, high acceptance among the majority of people in developing countries, relative inexpensiveness, less dependence to technology and economy. Among several traditional and complementary medicines, herbal Medicine and acupuncture are from prevalent methods. Side effects of these methods have been published in valid international journals (11). Herbal Medicine as a complementary method has been applied in many communities, from thousands of years ago. WHO has estimated that an impressive 80 % of the world population in use medicinal plants for treatment that these states are more in developing countries and are less in developed countries (12).

Ginger with scientific name of Zingiber Officinal has long history. This plant has been used as a drug from ancient time and is recorded in ancient medical texts in China, old Greece, Rome, and Arabia. Ginger is effective in treatment of nausea and vomiting, and has no specific side effect (13). Ginger rhizome is its root that contains many active biological compounds. Major pharmacological activities of ginger are related to its active compounds including gingerol and Shogaols. The effects of these combinations are anti-inflammatory, antiemetic, antipyretic, Anti-tussive, antihypertensive, anti-cancer, decreasing of prostaglandin, and the sedation of digestive problems. The effect of ginger products as an antiemetic is implemented by several mechanisms. For example, ginger and shogaols decrease the stomach contractions, but increase the activity of gastro intestinal tract (GIT). These combinations have anti-cancer effect and exert the effects of garbage scavenger against of the free radicals (14). Ginger compounds, including 6-Gingerol, 6-Shogaols, and Galanolactone, have shown anti HT5-receptor activities in guinea Pigileum and also Galanolactone acts as a competitive HT5 antagonist in ileum (15). Also ginger is listed as a food on the FDA’s “generally regarded as safe” list. Research studies have demonstrated ginger’s effectiveness against nausea associated with motion sickness, pregnancy, and surgery (10, 16).

Ozgoli, et al. (16) have done a research using ginger on 67 pregnant women (35 person as a control group and 32 person as intervention group) regarding effects of ginger capsules on pregnancy, nausea, and vomiting. They found that the ginger improves the nausea intensity and frequencies of vomiting of pregnancy (16). In the case of using ginger for patients undergone surgery, an study on 60 patients of laparoscopy surgery, conducted by Apariman, et al. (4) with the subject of “Effectiveness of ginger for prevention of nausea and vomiting after gynecological laparoscopy”, showed that nausea and vomiting after a surgery will be decreased noticeably in groups treated by ginger, compared to ones received placebo (4). In controlled trials by Chittumma et al. and Jenabi et al. is shown that ginger can be effective as vitamin B6 in controlling nausea and vomiting of pregnancy and there was no need for any additional therapy in ginger group (17, 18).

## 2. Objectives

Due to the growing surgical procedures in hospitals and nausea and vomiting being the most common complication after surgery and general anesthesia, considering to the fact that drugs, used to prevent nausea and vomiting, have side effects, this study was done with the aim measuring effects of ginger on postoperative nausea and vomiting.

## 3. Patients and Methods

This study is a randomized, double - blinded clinical trial with two groups (Ginger-Placebo) conducted in Khatam al Anbia hospital in Shahroud ,Iran, from March 2011 to August 2012. The research proposal was approved by the committee of ethics of Shahroud University of Medical Sciences. All participants were aware of the study goals, and they signed an informed consent prior to the study. We selected 160 patients, willing to participate in the study. Considering the formula (Matthews, John N.S. Introduction to Randomized Controlled Clinical Trials, 2nd Edition, Chapman and Hall/CRC, London 2006. PP. 25-39), α = 0.05 , β = 0.1 (CI = 95% and power = 90 %) and also regarding global and regional studies, the expected incidence of nausea of 30% and the expectation of being able to identify 10% difference in prevalence; The sample size was 79 patients in each group.[Fig fig8317]

**Figure 1. fig8317:**
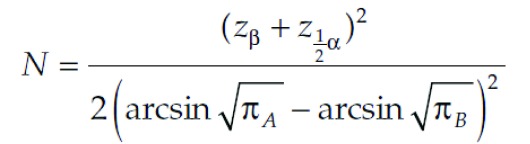
Equation

The inclusion criteria were: Lack of affliction of cancer, being 18-20 years old, ability to swallow capsules, platelet count above 100 thousand, Lack of afflictions of hepatitis and digestion system blockage, lack of pregnancy, Lack of receiving PRN or drugs cause vomiting, Lack of long time treatment with corticosteroid drugs, and having no experience of ginger sensitivity The exclusion criteria were using any substance or drug effective on nausea and vomiting. During the research 2 of the participants in placebo group were excluded from the research list. The first one removed from the list because of the severe vomiting, after using the capsules due to the surgery’s stress; and the second one had used high doses of a narcotic without informing the researcher. Thus the analysis was continued in accordance with the available data. This study was done on the basis of the block randomization (balance block random) with the four block method.

All of the patients admitted to hospital for surgery after reviewing inclusion and exclusion criteria were enrolled in study. For randomization of Participants, we used 4 sized blocks including 6 possibilities. In order to reach expected sample size we randomly chose possibilities and based on listed possibilities, Participants were allocated in A and B groups as follows: one of the researchers after being telephonically informed the presence of a case in hospital, based on prepared list placed the case in a group. After that explaining the purposes of the study, taking informed consent, giving coded capsules and gathering information was done by 2 trained nurses who did not know the nature of A and B capsules.

Participants in the intervention group received 4 ginger capsules and people in placebo group received 4 Placebo capsules. It is necessary to explain that ginger capsule, with Zintoma trade mark, consists of 250 mg ginger powder which was produced by Isfahan Gol daru and placebo consists of 250 mg ineffective powder (Chickpea powder). The shape, color, taste and fragrance of this powder were similar to ginger and both of them were provided by the same company. There was no routine protocol before surgery in this hospital. The patients swallowed capsules (A and B) with 30ml water, 1 hour before the surgery. The decision for type of anesthetic, method of using anesthetic, duration of anesthesia, and prescription of narcotic drugs during the surgery was made by anesthesiologist regarding type of surgery and condition of patients. According to the patients’ request, after the surgery, analgesics were prescribed and antiemetic drugs were also administrated after two times vomiting for each patient (without consideration of category in which the patients have been located).

The style of assigning scores, in accordance with VAS visual analogue scale, was explained for all of the patients. This criterion is composed from a 10 cm line (range: 0-10). Zero is assigned for no nausea and 10 shows severe nausea. Rating tool was as follows: zero related to no nausea, scores in the range 1-3 shows mild nausea, and scores in the ranges 4-6 and 7-9 related to moderate and severe nausea, respectively; too severe nausea is also shown with score 10. In similar studies the VAS tool was used for measuring abstract problems such as nausea (3, 9, 19, 20). Vomiting was also defined as severe gastrointestinal motility that leads to existing gastro intestinal contents out of mouth. Frequencies of retching were asked from patient and recorded.

Information obtained from patients included: Age, type and time of the surgery, demand for narcotics and analgesics after the surgery, type of anesthesia , duration of anesthesia, score of VAS visual analogue scale, and the periods of retching and vomiting have also evaluated. Two, four and 6 hours after surgery, intensity of patients’ nausea and vomiting was evaluated with using questionnaire and standard tools, by a researcher who wasn’t aware of the treatment regime (A or B). It should be noted that all of the participants of the project have been questioned about probable side effects such as stomach ache, heartburn, asthma, and insomnia; these side effects and their frequencies have recorded, if any. Quantitative data is shown as an average ± standard deviation and qualitative data is also demonstrated as frequency and percentage. Finally, the data was analyzed statistically by using SPSS version 16 and STATA version 11: Statistical tests of chi-square, t test, Mann-Whitney U and GEE test were used. Irct ID: IRCT138805202329N1.

## 4. Results

160 patients, 97 male (60.6%) and 63 female (39.4%) were studied. 45 patients(55.6%) in intervention group and 52 patients (56.8%) in placebo group were male. participants in the two groups were analyzed for characteristics such as age, systolic blood pressure, anesthesia duration and type of surgery and no statistically significant difference was seen (Table 1). 

The average nausea score 2 hours after surgery was lower for ginger using patients (2.9 ± 2.1) than placebo group (3.5 ± 1.9). the average nausea score four and six hours after surgery was lower for ginger using patients than placebo group but no statistically significant difference was seen in any hour. the average numbers of nausea in ginger using patients was lower than placebo group two, four and six hours after surgery but this difference was marginally significant (P = 0.053) 2 hours after surgery (Table 2). 

As demonstrated in Table 2, nausea scores and numbers of nausea increased during 2nd to 6th hour after surgery in both groups with exception of 4th hour after surgery in placebo group having a mean nausea scores lower than the 2nd hour. 

Nausea intensity in ginger using patients based on given visual scores was not statistically significant different from placebo group (Table 3). The number of vomiting in two groups was also compared. Majority of patients, 76 person in ginger group and 70 patients of placebo group did not vomit in first 2 hours. Four and six hours after surgery in respective order, 76 patients (93.8%) and 81 ones (100%) in ginger group and 77 person (97.5%) and 78 patients (98.7%) in placebo group did not vomit. the maximum number of vomiting was reported in only just one patient and 2 hours after surgery. None of patients reported the side effects of given medications. 

**Table 1.** Comparing Ginger and Placebo Group Based on Primary Variables

Variable	Ginger Group	Placebo Group	P value
**Age, (y)**	37.1 ± 19.1	37.9 ± 20.1	0.8
**Systolic blood pressure (mmHg)**	112.9 ± 15.7	113.0 ± 19.9	0.96
**Diastolic Blood Pressure (mmHg)**	71.5 ± 8.9	72.0 ± 9.3	0.7
**Anesthesia Duration (minute)**	65.7 ± 40.3	68.0 ± 41.6	0.72
**Type of surgery**			0.21
Genitourinary system, (%)	21 (25.9)	24 (30.4)	
Orthopedics, (%)	23 (28.4)	26 (32.9)	
ENT, (%)	21 (25.9)	19 (24.1)	
Abdominal, (%)	16 (19.8)	10 (12.7)	

**Table 2.** Comparing Score and Number of Nausea in two Groups (Using ginger and Placebo) in 2, 4 and 6 hours After Surgery

Measuring hours	Ginger Group	Placebo Group	P value
**Nausea score**			
2 h	2.9 ± 2.1	3.5 ± 1.9	0.043
4 h	3.1 ± 2.1	3.4 ± 1.9	0.405
6 h	3.4 ± 2.1	3.7 ± 2.3	0.395
**Number of nausea**			
2 h	1.5 ± 1.1	1.77 ± 0.98	0.053
4 h	1.87 ± 1.1	2.02 ± 1.1	0.383
6 h	2.01 ± 1.1	2.22 ± 1.3	0.259

**Table 3.** Comparing Nausea Intensity Between two Groups in Different Hours After Surgery

Measuring Hours	Group	Nausea Intensity					P value
		No Nausea (%)	Mild (%)	Moderate (%)	Severe (%)	Very Severe	
**2nd h**	ginger	7 (8.6)	45 (55.6)	24 (29.6)	3 (3.7)	2 (2.5)	0.26
	placebo	6 (7.6)	37 (46.8)	30 (38)	6 (7.6)	0 (0)	
**4th h**	ginger	7 (8.6)	45 (55.6)	23 (28.4)	5 (6.2)	1 (1.3)	0.66
	placebo	6 (7.6)	42 (53.2)	25 (31.6)	5 (6.3)	1 (1.3)	
**6th h**	ginger	11 (13.6)	30 (37)	33 (40.7)	6 (7.4)	1 (1.2)	0.63
	placebo	6 (7.6)	35 (44.3)	28 (35.4)	8 (10.1)	2 (2.5)	

**Table 4.** Factors Effecting Number and Score of Postoperative Nausea using GEE Model

	(b)^a^		P -value		[CI:95%-Interval]	
	Nausea Score	Number of Nausea	Nausea Score	Number of Nausea	Nausea Score	Number of Nausea
**Administrating Capsules (Ginger or Placebo)**	-0.497	-0.256	0.052	0.034	-0.999 – 0.004	-0.493 – -0.019
**Administrating metoclopramide (2nd hour)**	2.450	0.817	0.002	0.025	0.937 – 3.963	0.102 – 1.531
**Administrating metoclopramide (4th hour)**	3.387	2.081	0.005	<0.001	1.031 – 5.742	0.968 – 3.194
**Constant**	3.470	1.972	<0.001	<0.001	3.114 – 3.826	1.804 – 2.140

^a^ Abbreviations: b; B Coefficient

In order to better analyzing the effect of intervention on number and score of nausea; administration of metoclopramide was considered in GEE model. As you can see the number and score of nausea is significantly greater in placebo, 2nd hour metoclopramide and 4th hour metoclopramide group (Table 4).

Due to limited number cases for administration of 6th hour metoclopramide (2 cases), this variable was not considered in GEE model.

In our GEE model the outcome variables can be estimate by following formula like a multiple linear regression model:

Y = β0 + β1X1 + β2X2 + β3X3

According to Table 3 for numbers of nausea we have: 

N. of nausea = 1.97 + -0.256 (ginger/placebo) + 0.817 (metoclopramide 2) + 2.081 (metoclopramide 4)

Due to dichotomous classification of independent variables (0 and 1), it is obvious that numbers of nausea is significantly 0.256 times reduced in ginger using group (P-value=0.034).

Similarly as demonstrated in Table 3, intensity of nausea is also 0.497 times lesser in ginger group; although this effect is marginally significant (P-value = 0.054). 

## 5. Discussion

Postoperative nausea and vomiting is one the most annoying problems caused by anesthesia. Although preventing use of anti nausa drugs were considered but side effects of such medications were always debated. Some of predisposing factors for postoperative nausea and vomiting include age, sex, anxiety, long duration of surgery, use of sedatives, anesthesia etc. Ginger is an ancient plant of many Asian countries. Ginger affects digestive movements, acids absorption and block of enterogastric reflexes and the resulting nausea and vomiting. Although antiemetic effects of ginger were long time detected but its effective dose is not determined yet.

In this study the ginger dose was 1 g that considered being safe and having no side effects. Ryan et al. studied 576 patients and concluded that 0.5 to 1 gram of ginger is significantly effective in decreasing intensity of nausea in acute phase of chemotherapy in adult cancer patients (21). VAS scores in this study were lower in ginger group than placebo in 2nd hour after surgery; in addition number of nausea in intervention group in that hour was less than placebo group. Opposing our results, Apariman et al. (4) concluded that nausea average score based on VAS in 2nd hour after surgery was lower in both groups than 6th hour after surgery. They assumed that pain and moving the patient from recovery to ward were the probable underlying causes of such an observation. They also claimed that using sedative drugs like pethidine, 2 h after surgery can cause nausea and vomiting. In six hours after surgery nausea score of pethidine group was compared to other group and no difference was seen in average scores of the two groups, but the nausea score of pethidine using group was significantly lower in ginger group than placebo (4). It was concluded that ginger minimize vomiting side effect of opioids.

Based on this study prevalence of vomiting in two groups in measured hours did not have statistically significant difference and ginger was effective in preventing nausea 2 hours after surgery. Also other studies have reported antiemetic effects of ginger (4, 9, 14-16). In meta-analysis conducted by Chaiyakunapruk et al. including 5 controlled trials having 363 patients and studying the effect of ginger on postoperative nausea and vomiting, it is shown that ginger is more effective than placebo (22, 23). In Pongrojpaw et al. study a significant difference in incidence of nausea between ginger and placebo group was also observed in 2nd and 4th hour after surgery but 24 h after surgery results were the same in 2 groups. Incidence of vomiting was less in ginger group but it was not statistically significant (23). Nanthakomon et al. (23) reported that ginger reduced incidence of postoperative nausea and vomiting in 2 to 4 hours after surgery (20). The results of this study show that patients who used placebo had a higher rate and score of nausea and also more Plasil was administrated for these patients. In concomitance with this study, Sontakke et al. research demonstrated that ginger has the same efficacy as Plasil in controlling nausea (21). 

It should be noted that in mentioned study there was only a single evaluation time which can affect the results. Leopold et al. also reported zero anti emetic effect of ginger which can be due to use of lower doses that did not result in therapeutic levels (24). In this and any other studies, ginger appeared to be side effects free (13-15, 17, 24).

One of the limitations to this study is non-injectability of ginger and enteral form of administration can be problematic in aesthesia process, so we were forced to use small doses of ginger. In addition, not matching the surgery types could affected the results.

Based on the results of this study, ginger was effective in prevention of nausea hours after surgery and no side effects were observed in this dose of ginger. It appears that ginger can be used as a safe drug for controlling nausea. Due to paradoxical results of different studies, we suggest conducting more studies and with higher doses of ginger and for longer surgeries.
